# Calcium Hydroxyapatite Combined with Photobiomodulation for Bone Tissue Repair: A Systematic Review

**DOI:** 10.3390/ma18051120

**Published:** 2025-03-01

**Authors:** Camila Pascoal Correia dos Santos, Paola Tatiana Espinosa Cruel, Daniela Vieira Buchaim, Marcelo Rodrigues da Cunha, Edilson Ervolino, João Paulo Mardegan Issa, Maria Angelica Miglino, Rogerio Leone Buchaim

**Affiliations:** 1 Graduate Program in Applied Dental Sciences, Bauru School of Dentistry, University of Sao Paulo, Bauru 17012-901, Brazil; camila.pcs@usp.br (C.P.C.d.S.); paolaespinosacruel@usp.br (P.T.E.C.); 2 Graduate Program in Anatomy of Domestic and Wild Animals, Faculty of Veterinary Medicine and Animal Science, University of Sao Paulo (FMVZ/USP), Sao Paulo 05508-270, Brazil; danibuchaim@alumni.usp.br; 3 Medical School, University Center of Adamantina (FAI), Adamantina 17800-000, Brazil; 4 Postgraduate Program in Health Sciences, Faculty of Medicine of Jundiaí (FMJ), Jundiaí 13202-550, Brazil; marcelocunha@g.fmj.br; 5 Department of Basic Sciences, School of Dentistry, São Paulo State University (UNESP), Araçatuba 16015-050, Brazil; e.ervolino@unesp.br; 6 Department of Basic and Oral Biology, Ribeirao Preto School of Dentistry, University of Sao Paulo, Ribeirao Preto 14040-904, Brazil; jpmissa@forp.usp.br; 7 Postgraduate Program in Structural and Functional Interactions in Rehabilitation, Postgraduate Department, University of Marilia (UNIMAR), Marilia 17525-902, Brazil; miglino@usp.br; 8 Postgraduate Program in Animal Health, Production and Environment, University of Marilia (UNIMAR), Marilia 17525-902, Brazil; 9 Department of Biological Sciences, Bauru School of Dentistry (FOB/USP), University of Sao Paulo, Bauru 17012-901, Brazil

**Keywords:** photobiomodulation, low-level laser therapy, hydroxyapatite, regenerative medicine, tissue engineering, bone regeneration, bone repair, hard tissue repair, calcium phosphate

## Abstract

Repairing hard tissues, such as bones, remains a significant challenge, especially in adverse clinical conditions. Calcium hydroxyapatite (CaHA), a calcium phosphate (CaP), has structural and chemical characteristics similar to the mineral structure of human bones and teeth, offering bioactivity and biocompatibility properties. Photobiomodulation (PBM) uses light to reduce inflammation and accelerate tissue healing. This systematic review analyzes the combination of CaHA and PBM from 25 studies extracted from the PubMed, Web of Science, and ScienceDirect databases, using the keywords “hydroxyapatite AND photobiomodulation”, “calcium hydroxyapatite AND photobiomodulation”, and “low-level laser therapy AND calcium phosphate.” All studies focused on bone regeneration, with no mention of soft tissue applications. The most commonly used calcium-based material was biphasic calcium phosphate (76%), a combination of CaHA and β-tricalcium phosphate, while 16% of the studies did not specify the brand or product used. With regard to PBM, the most commonly used wavelengths (48% of cases, with a tie of 24% for each) were infrared lasers at 808 nm and 780 nm, with 20% of studies not mentioning the brand or manufacturer. The results underscore the predominant focus on bone regeneration, highlighting the need for further investigations into soft tissue applications and the establishment of standardized protocols. The combination of CaHA and PBM shows promise in regenerative medicine and dentistry, although more research is needed to expand its experimental and clinical use.

## 1. Introduction

Fast, efficient, and safe tissue repair remains one of the biggest challenges of regenerative medicine, especially in less-than-ideal clinical situations, where cell regeneration is crucial for functional recovery. The search for innovative therapies has driven the development of research on bioactive materials that can act synergistically with cell stimulation techniques [1]. Among these materials, calcium hydroxyapatite (CaHA) stands out, with the chemical formula Ca10(PO4)_6_(OH)_2_ [2,3,4], which is a calcium phosphate (CaP) widely found in the bone structures of animals and humans [5]. Due to its remarkable versatility and extensive applications in various fields of health, calcium hydroxyapatite has become a significant focus of scientific research in recent years [6,7].

This biomaterial is highly valued for its biocompatibility, osteoconductive properties, minimal cytotoxicity, affordability, and ease of production, making it a leading option in regenerative medicine [8,9]. In addition, CaHA has demonstrated exceptional ability to promote vascularization and facilitate effective bone formation when applied to target areas, especially in combination with other biomaterials or advanced therapeutic modalities such as photobiomodulation (PBM) [10,11,12,13,14,15,16].

The search to mimic CaHA in laboratory settings has gained strength in the scientific literature, aiming to enhance its intrinsic benefits and maximize its potential in biomedical applications [8]. Initially focused on orthopedics and bone regeneration due to its ability to accelerate healing [17], CaHA has extended to the dental field, where it reinforces resin matrices to improve structural integrity [18,19]. In the aesthetic domain, CaHA is widely used for skin rejuvenation and collagen biostimulation, providing significant therapeutic value in these areas [17]. Studies also demonstrate that when CaHA is combined with other materials, it improves tissue repair, as seen in applications for burn treatments in dermatology [20,21].

To support its growing utility, researchers have developed some innovative methods to extract and reproduce CaHA; for example, the extraction of fish bones has shown promise as a sustainable and affordable alternative [2]. Meanwhile, complementary research on light amplification by stimulated radiation emission (LASER) technology has gained attention. This technique uses light at specific wavelengths to stimulate biological processes, and its application in low-level laser therapy (LLLT) has demonstrated significant benefits [22]. When applied appropriately, LLLT modulates inflammation, provides analgesic effects, and accelerates soft and hard tissue healing, further expanding the synergistic potential of combining CaHA with PBM for advanced regenerative therapies [10,11,23,24,25,26,27,28].

The combination of CaHA with PBM could represent significant advances in regenerative medicine, offering a more comprehensive and effective approach to tissue repair. The objective of this article is to fill this gap by providing a critical view of the combined application of CaHA and PBM in the repair of various types of tissues and exposing the parameters used. This review will be carried out with search and eligibility criteria, through the collection of data from the available literature on the application of these techniques in soft and hard tissues, with the aim of providing a comprehensive understanding of the advantages and limitations of this combination.

## 2. Materials and Methods

### 2.1. Protocol and Study Design

This systematic review was conducted in accordance with the Preferred Reporting Items for Systematic Reviews and Meta-Analyses (PRISMA) guidelines. The PICO strategy was employed as follows: population: patients or experimental models (animals) undergoing bone regeneration procedures using calcium hydroxyapatite (CaHA) and photobiomodulation (PBM); intervention: combination of CaHA and PBM for bone repair, assessing their effects on bone formation, inflammation reduction, and healing acceleration; comparison: use of CaHA alone or PBM alone versus the combined approach, evaluating differences in effectiveness; and outcome: improved bone healing, increased bone mineral density, and enhanced tissue organization in cases where CaHA and PBM were combined compared to isolated interventions.

### 2.2. Eligibility Criteria

After crossing the keywords, a sequence of criteria was applied to reach the final and ideal selection of manuscripts, starting with the reading of titles, abstracts, and finally, the full articles, adhering to the inclusion and exclusion criteria outlined below. The inclusion and exclusion criteria were as follows:

Inclusion:

- Studies on animals and humans;
- In vivo/in vitro studies;
- Case reports;
- Publications in English;
- Access to the full article.

Exclusion:

- No association with the healing process;
- Systematic review articles or book chapters;
- Unpublished abstracts;
- Theses or dissertations;
- Absence of the combined use of CaHA or calcium phosphate;
- Non-English languages or restricted access.

### 2.3. Literature Research

To ensure a comprehensive search for relevant studies, the search strategy incorporated a combination of keywords and terms related to calcium hydroxyapatite and photobiomodulation. Primary keywords included “hydroxyapatite”, “calcium hydroxyapatite”, “photobiomodulation”, “low-level laser therapy”, and “calcium phosphate”. The Boolean operators “AND” and “OR” were used strategically to combine these terms effectively, ensuring the inclusion of pertinent studies. The following is the search equation applied in the PubMed, Web of Science, and ScienceDirect databases. The last search was dated 12/03/2024.

PubMed/MEDLINE: (“hydroxyapatite”[tiab] AND “photobiomodulation”[tiab]) OR (“calcium hydroxyapatite”[tiab] AND “photobiomodulation”[tiab]) OR (“low-level laser therapy”[tiab] AND “calcium phosphate”[tiab]).

Web of Science: TS = (“hydroxyapatite” AND “photobiomodulation”) OR (“calcium hydroxyapatite” AND “photobiomodulation”) OR (“low-level laser therapy” AND “calcium phosphate”).

ScienceDirect: TITLE-ABSTR-KEY(“hydroxyapatite” AND “photobiomodulation”) OR TITLE-ABSTR-KEY(“calcium hydroxyapatite” AND “photobiomodulation”) OR TITLE-ABSTR-KEY(“low-level laser therapy” AND “calcium phosphate”).

### 2.4. Study Selection and Data Extraction

**Figure 1:**
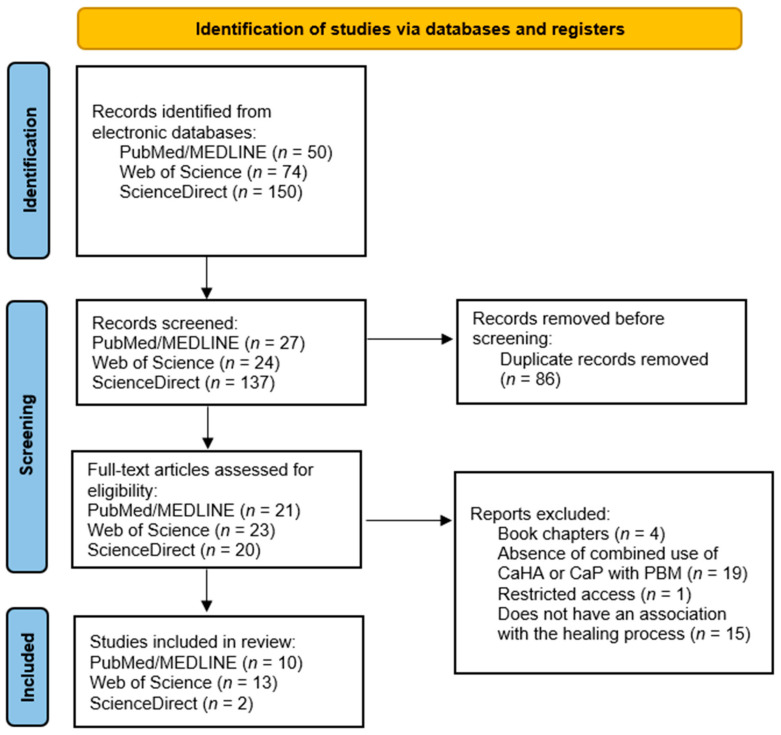
Articles identified in the databases and organized through the flow diagram.

The articles were read and analyzed thoroughly by two independent researchers (C.P.C.d.S. and P.T.E.C.) to ensure the rigorous application of the inclusion and exclusion criteria, minimizing the risk of bias. After reading, the data were collected and organized into tables by the authors and later compared. Any discrepancies were resolved after a final analysis based on the established criteria. Finally, a PRISMA flowchart was developed to represent the study selection process, as illustrated in Figure 1.

## 3. Results

In the search conducted across bibliographic databases, 50 articles were found on PUBMED/Medline, 74 articles on Web of Science, and 150 articles on ScienceDirect.

Of these, 40 articles from PUBMED/Medline, 61 articles from Web of Science, and 148 articles from ScienceDirect were excluded due to duplication or failure to meet the designated inclusion and exclusion criteria. In total, 25 articles were analyzed qualitatively and in detail.

**Figure 2:**
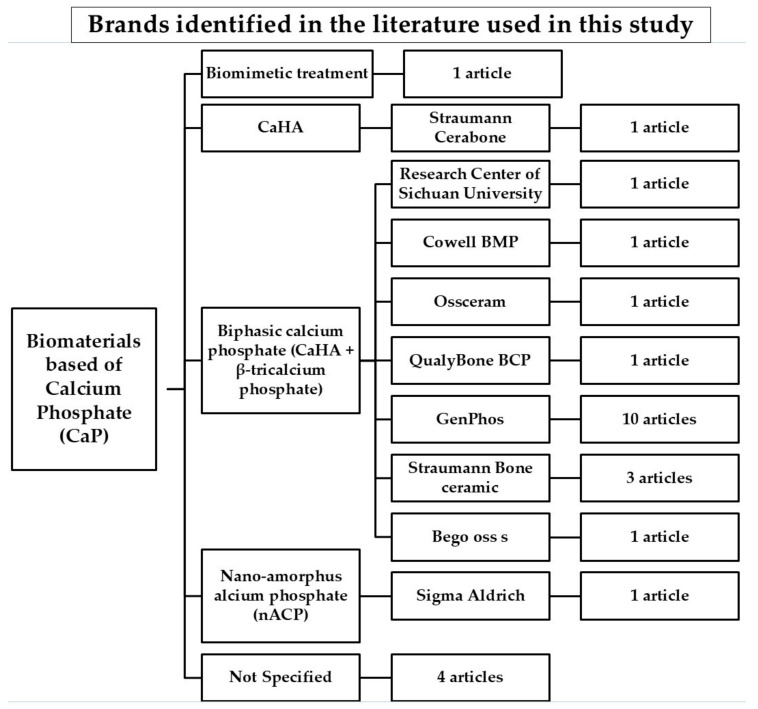
Brands identified in the scientific literature used in this study.

Regarding the commercial brands associated with CaHA, we found that GenPhos (Baumer^®^, Mogi Mirim, Brazil) was the most cited brand, present in 40% of the studies. Furthermore, 16% of the studies did not specify the commercial brand of the biomaterial used. It is important to mention that the most frequently found biomaterial was biphasic calcium phosphate, composed of CaHA and β-tricalcium phosphate. A detailed analysis of all the brands of the biomaterial used in each article is shown in Figure 2.

**Figure 3:**
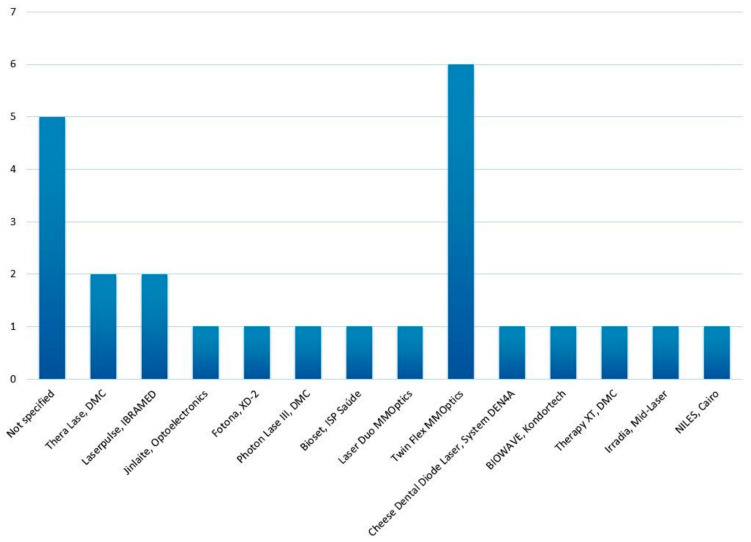
Distribution of commercial brands and manufacturers of light therapies used in the analyzed studies (*n* = 25).

According to the most frequently used commercial brands in PBM, it was observed that the Twin Flex brand (MMOptics^®^, Sao Carlos, Brazil) was employed in 24% of the studies. Additionally, 20% of the authors did not specify the brand or manufacturer. Below, in Figure 3, all the commercial brands and manufacturers used in each article are listed.

As stated earlier, the 25 articles are presented in Table 1, providing not only the PICO strategy (P: patient or problem; I: intervention; C: control; O: outcome) but also the references, information on the CaHA/CaP used and its objective, and details on the PBM protocol.

## 4. Discussion

The main objective of this article was to compile all the evidence available in the literature on the efficacy of combining a calcium-based biomaterial, such as calcium hydroxyapatite (CaHA), and photobiomodulation (PBM) as a regenerative therapy for hard tissues. The objective was to gather as much data as possible on application techniques, parameters, protocols, advantages, and potential limitations of this combination, focusing on aspects that have been little explored, especially outside the context of bone regeneration.

In previous studies, CaHA has been used as a filler biomaterial, highlighting its osteoconductive properties [32,33,35]. In addition, a distinct approach used CaHA to induce crystal deposition, allowing for the evaluation of laser efficacy in treating acute and chronic pain [29]. These different approaches underscore the versatility of CaHA in various clinical applications, reinforcing its essential role in tissue repair.

Table 1 shows different calcium-based biomaterials, such as pure hydroxyapatite (CaHA) [44] and the combination of CaHA and β-tricalcium phosphate (biphasic phosphate) [43], both of which aim to evaluate their efficacy in bone regeneration in experimental models. The first study [44] used pure hydroxyapatite combined with LED therapy (850 nm) in a dental alveolus model, demonstrating improved bone formation, with significant increases in bone density and volume, particularly at 30 days. The second study [43] investigated a biphasic material (60% HA and 40% β-TCP) combined with mesenchymal stem cells and a low-level laser (808 nm) to assess bone regeneration. This combination showed notable effects on bone mineralization, particularly during the early stages of regeneration.

Pure hydroxyapatite [44] was effective in enhancing bone density and structural organization, especially when combined with LED therapy. In contrast, biphasic phosphate (HA + β-tricalcium phosphate) [43], paired with mesenchymal cells and a laser, showed superior results in promoting early bone mineralization. All studies reported significant differences between treated and control groups (*p* < 0.05), highlighting the relevance of these approaches in bone repair.

Among the studies conducted by Pinheiro et al. using the combination of β-tricalcium phosphate with hydroxyapatite (CaHA) [16,32,34,36,38] in different animal models, such as rats and rabbits, two in particular [16,32] stand out for a more detailed comparative analysis, although other studies have also addressed this combination. In the experiment carried out with rats [16], a faster initial bone formation was identified, especially when combined with photobiomodulation, resulting in less inflammatory infiltration and a better organization of the connective tissue in the first weeks. On the other hand, in the research with rabbits [32], the findings showed a more accentuated bone maturation over time, with an increase in bone mineral density, although the initial formation occurred more slowly. This comparison reinforces the efficacy and consistency of the combination of CaHA and β-tricalcium phosphate as well as highlighting the careful approach of Pinheiro et al. in testing and validating the material in different experimental contexts, solidifying it as a promising alternative for bone regeneration.

The study by Allam et al. [48] was the only one that investigated the use of nano-amorphous calcium phosphate (nACP). The results showed that the laser alone promoted greater bone formation and mineral density, while the nACP showed slow resorption and bone formation limited to the defect margins. The combination of nACP and a laser showed no further improvements, suggesting that the material may have acted as a physical barrier, impairing light penetration and its biostimulatory effect. These results indicate that further studies are needed to determine the best therapeutic approach involving calcium-based biomaterials, exploring different formulations, concentrations, and combinations with complementary therapies. In addition, because nACP has been poorly investigated to date, further research may reveal ways to optimize its bioactive properties and its role in tissue engineering.

Despite these advantages, some studies have revealed important limitations. Although most studies show that calcium-based biomaterials associated with PBM have accelerated initial bone formation, their long-term effectiveness is still uncertain. Furthermore, one study [30] highlighted that external factors, such as exposure to smoking, can significantly compromise the regenerative efficacy of both CaHA and CaP, reinforcing the need to consider specific clinical variables.

**Figure 4:**
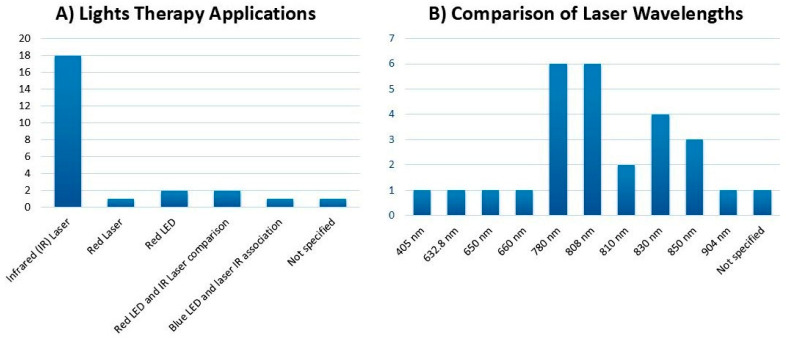
(**A**): The distribution of light therapies used in the studies analyzed, including infrared (IR) lasers, red lasers, red LEDs, and blue LEDs, both individually and in combination. (**B**): A comparison of wavelengths used in laser and LED therapies (*n* = 25), showing 405 nm (blue LED) and 632.8 nm (red He-Ne laser), alongside various infrared wavelengths.

Regarding the use of photobiomodulation as an adjuvant therapy during bone repair, the wavelengths used ranged from 405 to 904 nm, with 780 nm reported in six studies [13,32,34,35,36,38] as well as the 808 nm wavelength, also reported in six studies [15,31,39,40,41,43]. The infrared laser was predominant, appearing in 18 studies that focused exclusively on bone tissue, as shown in Figure 4. Among the remaining studies, a red laser was used in one experimental study on rats [29], two used light-emitting diodes (LEDs) as stand-alone devices [33,44] and two compared the effects of laser and infrared LED devices [36,38]. Notably, one study explored the combination of blue LED light and an infrared laser, producing promising results for the osteogenic differentiation of human dental pulp stem cells [46]. Furthermore, one study did not specify the type of laser and wavelength used.

LED technology, initially introduced in 1928, has been widely employed since the 1990s for the treatment of chronic and postoperative pain [49]. In the context of photobiomodulation (PBM), both LEDs and lasers have demonstrated efficacy as adjunctive therapies in soft and hard tissue healing despite their distinct characteristics and mechanisms [50]. Comparative data on these therapies, including their wavelengths and applications, are summarized in Figure 4.

On the other hand, Pinheiro et al. [32,36,38] and Soares et al. [13,35] demonstrated that the standardization of laser therapy parameters, including light types and wavelengths, significantly improved the consistency of the results, reinforcing the importance of uniform protocols. The interaction between CaHA and PBM has been studied to assess whether there is a synergistic effect, i.e., whether their combined use results in greater benefits compared to individual therapies. According to most of the reviewed articles, the simultaneous use of both therapies resulted in increased fibroblast proliferation and collagen secretion, leading to improvements in tissue organization and bone maturation [32,33,44].

Among the most widely used devices, the gallium-aluminum-arsenide (GaAlAs) laser was predominant, being considered the most effective due to its good tissue penetration and ability to promote bone mineralization [51,52,53]. Most of the studies in the table have proven its efficiency in accelerating the repair of bone defects and improving the quality of the mineral matrix. In addition to lasers, LED technology has also been investigated in some studies [38,44]; 850 nm LEDs showed potential under specific laboratory conditions. However, its lower intensity compared to lasers was pointed out as a limitation in more complex cases.

The application protocols varied widely among the studies, from daily sessions to 48 h intervals or three times a week, with energy doses between 4 J/cm^2^ and 20 J/cm^2^. This heterogeneity in the parameters made direct comparisons between the results difficult and became an obstacle to clinical standardization.

The combination of lasers with biomaterials, such as calcium hydroxyapatite (CaHA) and biphasic calcium phosphate (BCP), has shown promise. Most of the articles in the table indicated that this association enhances initial bone regeneration. In contrast, one clinical study [19] observed no significant additional benefits when combining CaHA with laser in maxillary sinus augmentation procedures, highlighting that the effects of the therapy may depend on the specific clinical context.

**Figure 5:**
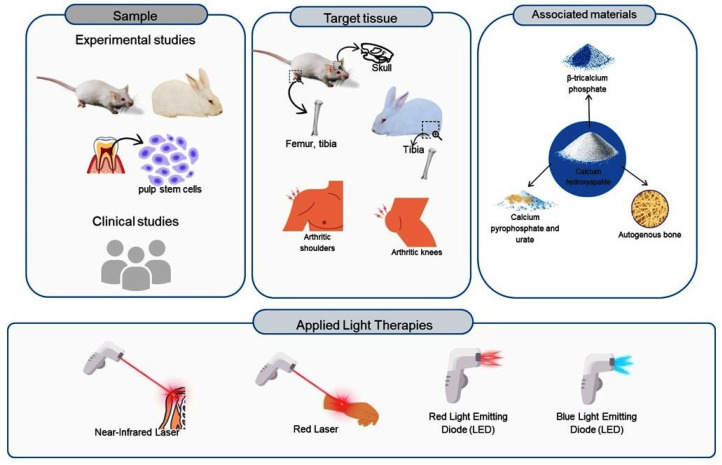
A representation of samples, target tissues, biomaterial associations, and types of LEDs and lasers covered in the article.

It is also important to note that the experimental studies have focused on short-term outcomes, specifically on early bone formation. There are still no medium- and long-term studies available on the combination of CaHA with PBM, especially regarding bone maturation and remodeling. This limitation compromises the more comprehensive evaluation of therapeutic effects over time. Based on the approach discussed so far, the diagram in Figure 5 illustrates the samples, target tissues, biomaterial associations, and LED and laser types used in each article reviewed in this study.

Although the use of CaHA is well established in regenerative medicine and bone tissue dentistry [54,55,56], where it is considered effective, like in terms of osteoconduction and bone regeneration, especially when combined with other biomaterials such as copper and lithium [52], polylactic acid [53], and the administration of multiple drugs [42], there are few studies on its use in soft tissues, like the skin [17]. Preliminary studies suggest that this biomaterial may also promote wound healing [54,55,56] by increasing the migration and deposition of macrophages and fibroblasts at the wound site [57] and reducing pro-inflammatory cytokines, thereby creating an environment conducive to tissue regeneration [58]. However, the literature in this area is scarce, and the scarcity of studies makes it difficult to understand the potential of CaHA in soft tissues.

Regarding limitations, one of the biggest challenges in integrating calcium hydroxyapatite (CaHA) or calcium phosphate (CaP) and photobiomodulation (PBM) therapies lies in the resource demands required for their application, including financial investments, advanced technology, and the overhead associated with adopting these innovative approaches. While advances in CaHA synthesis are aimed at reducing production costs, achieving medical-grade quality and specific particle sizes suitable for regenerative applications remains a complex and expensive process. Similarly, the acquisition of high-performance PBM devices, such as lasers and LEDs, involves a significant financial investment, particularly in environments with limited funding. Additionally, the lack of standardized PBM protocols often requires extensive experimentation and adjustments, leading to increased time and material costs. These factors collectively limit the widespread implementation of these therapies, especially in regions with restricted access to advanced medical technologies. Addressing these barriers will require cost-effective innovations and ongoing research efforts to refine techniques while ensuring therapeutic efficacy.

**Figure 6:**
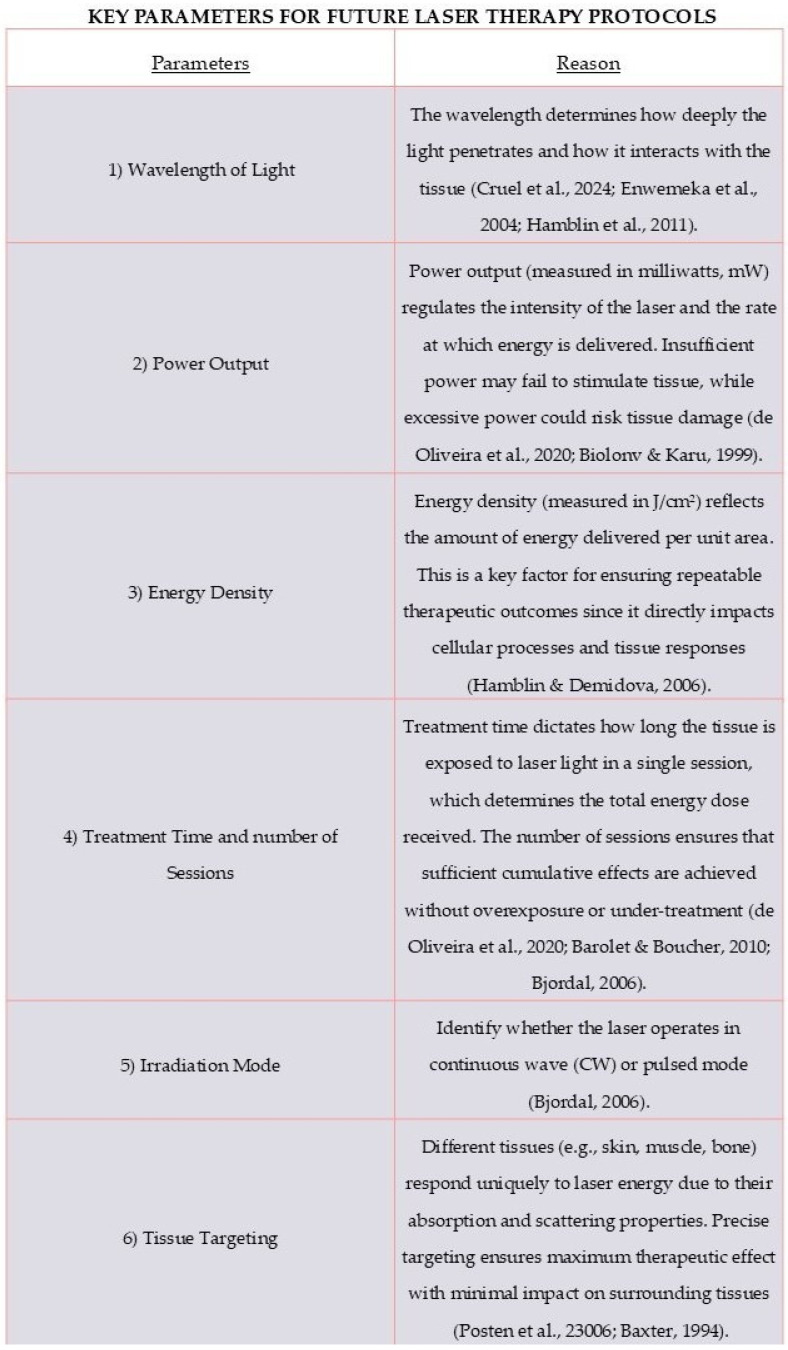
This table provides a framework to assist researchers and clinicians in developing reliable and efficient laser therapy protocols, ensuring consistency and enhancing clinical results [59,60,61,62,63,64,65,66].

Regarding suggestions of future protocol, the authors consider the development of standardized protocols a crucial factor for the reproducibility of future research. Standardized protocols are essential parameters that directly influence tissue responses and therapeutic effects. Below, we describe the most important parameters to consider when designing future laser therapy protocols and explain their relevance to achieving effective treatment outcomes (Figure 6).

## 5. Conclusions

This review highlights the potential of combining CaHA and PBM in tissue repair. The studies analyzed show that this combination not only increases the biocompatibility and osteoconductive capacity of the material but also enhances the effects of PBM in accelerating healing and reducing inflammation. CaHA is already widely recognized for its fundamental role in bone regeneration, while PBM is well established for its therapeutic properties. The synergistic interaction between the biomaterial in question and PBM has revealed new mechanisms that can optimize clinical results, especially in scenarios that require both structural replacement and functional recovery in a shorter period.

The information compiled has important implications for clinical practice, suggesting that the combination of CaHA and PBM could become a standard in regenerative therapies, with the potential to significantly improve patient recovery and functionality. However, there are still gaps in the literature, particularly regarding the standardization of laser therapy protocols and the application to the skin, emphasizing the need for more clinical research to establish clear and effective guidelines. This review aims not only to clarify the benefits of this approach but also to encourage further research that could advance regenerative therapies.

## Figures and Tables

**Table 1:** Articles selected according to the established criteria.

Biomaterial	Target Tissue Bone Sites	Subject	Laser Type	Laser Specifications	Duration	Limitations	Results	References
CaHA Crystals	Joints of the hind limbs	Female Suquia rats, ≈0.44 lb	He-Ne (632.8 nm) l	6 mW, energy density 8 J/cm^2^, direct use	Daily laser irradiation for 21 days	Limited model for replicating humans	PBM matched diclofenac in acute arthritis and excelled in chronic cases.	Soriano et al., 2006 [29]
Biphasic calcium phosphate (CaHA + β-tricalcium phosphate)	Femur	Male and female Wistar rats, ≈0.66 lb	Low-intensity laser (LPBM—λ 830 nm)	40 mW; continuous, spot size 0.6 mm; dose per point: 4 J/cm^2^; dose per session: 16 J/cm^2^; total dose: 112 J/cm^2^	Repeated 15 and 30 days	Preliminary results; difficulty in understanding the precise mechanism of biomodulation	PBM boosted bone repair and GBR with CaHA improved early healing.	Pinheiro et al., 2009 [16]
Biphasic calcium phosphate (CaHA + β-tricalcium phosphate)	Femur	Female Wistar rats	Gallium-aluminum-arsenide diode laser, 904 nm	100 mW, 5 J/cm^2^, total dose 20 J/cm^2^	3 times a week for 8 weeks	Prolonged exposure to smoke, limited LLLT protocols	Smoke impairs osseointegration; LLLT and HA limited efficacy.	Franco et al., 2013 [30]
Biphasic calcium phosphate	Calvaria	Male Wistar rats, ≈0.66 lb	Diode laser, 808 nm	0.1 W, 4 J/cm^2^ per session	3 days a week, for 2 weeks	Short period, lack of clinical validation.	Laser and ozone therapies increased bone formation; ozone was more effective.	Kazancioglu et al., 2013 [31]
Biphasic calcium phosphate (CaHA + β-tricalcium phosphate)	Tibia	Male adult New Zealand rabbits with a mean, 4.4 lb	Infrared laser (λ 780 nm)	50 mW. continuous Wave (CW), spot size 0.5 cm^2^. Dose 16 J/cm^2^ (4 × 4 J/cm^2^ per point)	Alternate days for 2 weeks	Preliminary results require clinical validation. Short evaluation period (30 days)	CaHA and PBM improved bone healing by increasing CaHA deposition and reducing organic components.	Pinheiro et al., 2013 [32]
Biphasic calcium phosphate (CaHA + β-tricalcium phosphate)	Tibia	Wistar rats, 1.10 lb	Infrared Light Emitting Diode (LED) (λ 850 ± 10 nm).	150 mW, continuous wave, spot size 0.5 cm^2^. 16 J/cm^2^ per application	Every 48 h for 15 days	Anemia affected graft integration; further research on inflammation needed. Short evaluation period (30 days).	LED phototherapy enhanced CaHA and improved bone repair in anemic conditions.	de Castro et al., 2014 [33]
Biphasic calcium phosphate (CaHA + β-tricalcium phosphate)	Femur	Male Wistar rats, ≈0.66 lb	Diode laser (λ 780 nm)	70 mW; continuous, spot size 0.4 cm^2^; dose/session 20 J/cm^2^; total dose 140 J/cm^2^	48-h intervals for two weeks	Non-critical size defect; limited to preclinical model	The Laser + Biomaterial group showed improved bone healing.	Soares et al., 2014 [13]
Synthetic biphasic microgranular CaHA (70% HA, 30% β-tricalcium phosphate)	Tibia	Male New Zealand rabbits, ≈4.4 lb	Infrared laser (λ780 nm)	50 mW; continuous, spot size: 0.5 cm^2^; Dose/session: 16 J/cm^2^; Total dose: 112 J/cm^2^	48-h intervals for two weeks	Short observation period; limited to a rabbit model.	Laser with graft and GBR improved bone repair in irradiated groups.	Pinheiro et al., 2014 [34]
Synthetic biphasic CaHA (70% HA, 30% β-tricalcium phosphate)	Tibia	Male Wistar rats, ≈0.66 lb	Diode laser (λ 780 nm)	70 mW; continuous, spot size 0.4 cm^2^; dose/session 20 J/cm^2^; total dose 140 J/cm^2^	48-h intervals for two weeks	Non-critical size defect; findings need further validation in clinical settings.	Laser and graft with GBR enhanced bone repair in irradiated groups.	Soares et al., 2014 [35]
Synthetic biphasic microgranular CaHA (70% CaHA, 30% β-tricalcium phosphate)	Femur	Male Wistar rats, ≈0.66 lb	Diode Laser (780 nm) or LED (850 nm)	Laser: 70 mW, spot size 0.4 cm^2^; LED: 150 mW, spot size 0.5 cm^2^; Dose/session: 20 J/cm^2^; Total dose: 140 J/cm^2^	48-h intervals for two weeks	Non-critical size defect; results specific to preclinical model	Synthetic CaHA + Beta-TCP graft improved bone repair, with or without Laser/LED.	Pinheiro et al., 2014 [36]
Biphasic calcium phosphate (CaHA + β-tricalcium phosphate)	Femur	Male Wistar albino rats, ≈0.55 lb	Gallium-aluminum-arsenide diode laser (λ 810 ± 10 nm)	300 mW, Point irradiation: 12 J/cm^2^ Mode: Not specified	3 times per week for 4 weeks	No significant differences between ozone and LLLT; further studies are needed.	At week 8, the PBM group showed increased osteocalcin and bone formation. Further studies are needed.	Hilal et al., 2015 [37]
Synthetic biphasic microgranular CaHA (70% HA, 30% β-tricalcium phosphate)	Tibia	Male Wistar rats, ≈0.66 lb	Diode laser (λ 780 nm); LED phototherapy (λ 850 ± 10 nm)	70 mW, 20 J/cm^2^/session, applied at 4 points (5.1 J/cm^2^/point). LED: 150 mW, 20 J/cm^2^, applied at 1 point over the defect.	Every 48 h for 2 weeks; evaluation at 15 and 30 days post-treatment.	Short follow-up (30 days); LED phototherapy less effective in bone maturation.	PBM enhanced bone repair in grafted defects, boosting CaHA deposition.	Pinheiro et al., 2017 [38]
Combination of 50% autogenous bone (AB) and 50% CaHA	Maxillary sinus floor bone	Humans, 48.12 years (20–60 years). Exclusion: smokers, uncontrolled systemic/local issues, prior head/neck radiotherapy.	Gallium-Aluminum-Arsenide (GaAlAs) laser (λ 830 nm)	40 mW, continuous mode irradiation at 4 points around the sinus cavity (5.32 J/point, 0.57 W/cm^2^).	No further applications during the 6-month follow-up.	Small sample size; results limited to 6 months; no subjective clinical data (e.g., postoperative comfort).	No differences in vital bone or immunohistochemistry; PBM sped up remodeling but not formation.	Theodoro et al., 2018 [19]
Coagulum, deproteinized bovine bone, and biphasic ceramic	Mandibular ramus region	Male Holtzman rats (*Rattus norvegicus*), ≈0.55 lb	Gallium-Aluminum-Arsenide (GaAlAs) laser (λ 808 nm)	100 mW, 1 J/point, 4 J/session, total 28 J. Beam diameter: 600 µm, fluence: ~354 J/cm^2^	Every 48 h for 13 days	Mechanical properties of repaired tissue not assessed; long-term effects beyond 90 days not evaluated.	PBM increased tissue mineralization and bone formation, especially after 90 days.	De Oliveira et al., 2018 [15]
Calcium phosphate	Tibia	Male New Zealand rabbits, ≈7.7 lb	GaAlAs, 808 nm, infrared laser	100 mW, continuous, 4 J/cm^2^ per point, 16 J/cm^2^ per session, total dose 122 J/cm^2^	Immediately after surgery, every 48 h for 7 days.	Animal model, short period (6 weeks), limited generalization.	CaP + LLLT accelerates initial osseointegration and improves fixation.	Do Prado et al., 2018 [39]
Bovine bone graft	Calvaria	Wistar rats, average weight ≈0.66 lb	Gallium-Aluminum-Arsenide (GaAlAs) laser (808 nm)	450 mW, continuous, 18.9 J per session Fluence: 24.075 J/cm^2^	Every 48 h for 14, 21, and 30 days	Small sample size; short-term effects; possible xenograft shielding of laser; results specific to rat calvarial defects.	PBM was most effective at 14 days, with reduced effects by 21 and 30 days	Luca et al., 2020 [40]
Deproteinized bovine bone and biphasic ceramic composed of CaHA and β-tricalcium phosphate	Tibia	Male Holtzman rats, ≈0.66 lb	Gallium-Aluminum-Arsenide (GaAlAs) laser (808 nm)	100 mW, ~0.60 mm diameter, continuous wave, 1 J per point, 4 points irradiated per session (total 4 J/session). Fluence: ~354 J/cm^2^ per point.	Every 48 h for 13 days	Laser penetration is limited by tissue thickness; LLLT timing and osseointegration need optimization.	PBM enhanced implant osseointegration in grafted areas, boosting bone formation and maturation proteins.	De Oliveira et al., 2020 [41]
Biphasic calcium phosphate	Calvaria	Male Wistar rats, 0.55 lb	GaAlAs (gallium-aluminum arsenide), 830 nm	30 mW; continuous, dose per point 6.2 J/cm^2^, dose per session 24.8J/cm^2^	Immediately after surgery and three times a week until euthanasia.	The interaction between PBM and the materials was not thoroughly investigated.	PBM and biphasic calcium phosphate favored bone regeneration.	Della Colleta et al., 2021 [42]
Biphasic calcium phosphate	Calvarial Periosteum	-Human BMSCs and human umbilical vein endothelial cells (HUVECs) -C57BL/6 female mice	GaAlAs, 808 nm	-In vitro: 40 mW; 4.5 J/cm^2^; 40 mW; dose per point 1.8 J/cm^2^, irradiation area 4 cm^2^	Immediately after surgery, every day until euthanasia	No analysis of long-term clinical viability.	PBM improved angiogenesis and osteogenesis.	Bai et al., 2021 [43]
100% pure hydroxyapatite	Dental alveolus (mandible)	Male Wistar rats, 0.66 lb	LED (850 nm)	100 mW, continuous, 2.8 cm^2^, 60 s of exposure, 35.7 mW/cm^2^ of irradiance, total: 48 J	15 days of irradiation with evaluations at 15 and 30 days	LED and biomaterial protocols need standardization for consistent, safe bone repair use.	LED and biomaterial improved bone maturation, density, and inflammation control.	Dalapria et al., 2022 [44]
Hydroxyapatite/tricalcium phosphate (BCP) and heterologous fibrin biopolymer	Calvaria	Male Wistar rats, ≈0.55 lb	GaAlAs laser (830 nm)	30 mW, continuous, 258.6 mW/cm^2^, energy density 6.2 J/cm^2^, beam area 0.116 cm^2^, total 2.9 J	3 times per week until euthanasia at 14 and 42 days	Further studies are needed optimize PBM parameters for effective bone repair with biomaterials.	PBM, especially with biomaterials, enhanced bone formation, indicating a synergistic effect in repair.	Reis et al., 2022 [10]
Biphasic calcium phosphate	Calvaria	Male Wistar rats, 0.66 lb	GaAlAs, 660 nm	30 mW, continuous, total dose 45 J/cm^2^	Single trans-surgical application	No analysis of long-term clinical viability.	PBM alone accelerated bone regeneration.	De Marco et al., 2022 [45]
Culture of human dental pulp stem cells (hDPSCs) with biphasic calcium phosphate	Subcutaneous implant in nude mice	Human dental pulp stem cells	Blue light and infrared LEDs LED (405 nm) NIR laser wavelength (810 nm)	Blue light: 1.2, 3.6, 12.0 mJ/cm^2^; NIR light: 87.8, 85.4, 77.0 mJ/cm^2^ for respective groups	Daily radiation for 5 weeks	Requires optimization of blue light dose to avoid cytotoxic effects while maximizing osteogenesis	Blue light with NIR boosted osteogenic differentiation, alkaline phosphatase, and calcium deposition more than NIR alone.	Kim et al., 2023 [46]
Biphasic calcium phosphate	Femur	Pomerian, ≈7.2 lb	Not specified	Not specified	Not specified	Exclusive use of a single clinical case	Accelerated bone union and vascularization.	Cho et al., 2023 [47]
Nano-amorphous calcium phosphate	Tibia	Male New Zealand rabbits, ≈7.7 lb	Diode laser, 650 nm	150 mW, continuous, 12 J/cm^2^	7 sessions spread over 2 weeks	Graft barrier may have reduced laser effectiveness.	Isolated laser increased bone density and healing.	Allam et al., 2024 [48]

List of Abbreviations Appearing in the Table: AB—autogenous bone; β-TCP—Beta-Tricalcium Phosphate; CaHA—calcium hydroxyapatite; CaP—calcium phosphate; GaAlAs—gallium-aluminum-arsenide; GBR—guided bone regeneration; HA—hydroxyapatite; hDPSCs—Culture of human dental pulp stem cells; LED—light-emitting diode; mW—Milliwatt; nm—Nanometer; PBM—photobiomodulation; BMSCs—bone marrow mesenchymal stromal cells; HUVECs—human umbilical vein endothelial cells.

## Data Availability

No new data were created or analyzed in this study.
